# Pioglitazone plus (−)‐epigallocatechin gallate: a novel approach to enhance osteogenic performance in aged bone marrow mesenchymal stem cells

**DOI:** 10.1002/2211-5463.70175

**Published:** 2025-12-05

**Authors:** Ching‐Yun Chen, Jia‐Ci Jhang, Chun‐Yi Peng, Quốc Cường Nguyễn, Feng‐Huei Lin, Hung‐Ming Wu, Shankung Lin

**Affiliations:** ^1^ Department of Biomedical Sciences and Engineering National Central University Taoyuan Taiwan; ^2^ Institute of Biomedical Engineering and Nanomedicine National Health Research Institutes Miaoli Taiwan; ^3^ Institute of Biomedical Engineering, College of Medicine and College of Engineering National Taiwan University Taipei Taiwan; ^4^ Department of Neurology Chung Shan Medical University Taichung Taiwan; ^5^ Graduate Institute of Acupuncture Science China Medical University Taichung Taiwan; ^6^ Translational Medicine Center Chung Shan Medical University Hospital Taichung Taiwan; ^7^ Graduate Institute of Biomedical Sciences China Medical University Taichung Taiwan

**Keywords:** advanced tissue maturation, bioengineered bone constructs, osteogenic enhancement, regenerative medicine

## Abstract

Previously, we reported that netoglitazone, a thiadolidinedione, enhanced both adipogenesis and osteoblastogenesis, and that fatty acid synthase knockdown could selectively repress the adipogenic effect. Here, molecular evidence further demonstrated that pioglitazone enhanced osteoblastic differentiation at least through the protein kinase A/glycogen synthase kinase 3β signaling. (−)‐Epigallocatechin gallate (EGCG), a fatty acid synthase inhibitor, selectively retained pioglitazone's pro‐osteoblastic effect. Cultures of aged human bone marrow mesenchymal stem cells (bmMSCs) in alginate scaffolds revealed that pioglitazone and EGCG cooperatively enhanced osteoblastic differentiation, biological apatite production, and bone‐like tissue maturation. These findings demonstrate that the combination of pioglitazone and EGCG is a promising strategy to enhance osteogenic performance in aged bmMSCs for the development of advanced bone graft materials.

## Graphical abstract

Aged human bmMSCs are seeded in the scaffold. Osteoblastic induction can slightly increase cell's bone‐forming activity to produce bone‐like tissues, shown as the sporadic xylenol orange‐stained spots (the lower left image). Notably, pioglitazone plus EGCG co‐treatment dramatically increases cell's bone‐forming activity and bone‐like tissue production (the lower right image).

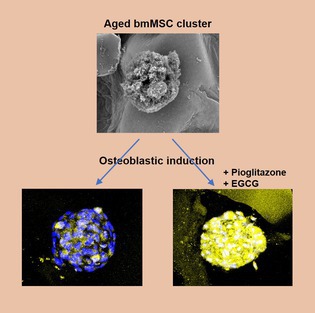

**Abbreviations**
ALP: alkaline phosphatase
AR: adenosine receptor
bmMSC: bone marrow mesenchymal stem cell
cAMP: cyclic adenosine monophosphate
CPC: cetylpyridinium chloride
ECM: extracellular matrix
EGCG: (−)‐epigallocatechin gallate
FASN: fatty acid synthase
FT‐IR: Fourier transform infrared spectroscopy
GSK3β: glycogen synthase kinase‐3β
IBMX: isobutylmethylxanthine
MCC‐555: netoglitazone
PKA: protein kinase A
TZD: thiazolidinedione
XRD: X‐ray diffraction
ZNF521: zinc‐finger factor 521

Thiazolidinediones (TZDs), such as pioglitazone, have been utilized to enhance insulin sensitivity in type 2 diabetes by activating peroxisome proliferator‐activated receptor γ (PPARγ), which regulates glucose homeostasis [1, 2, 3]. However, chronic TZD administration has been associated with adverse effects, notably bone loss, due to PPARγ's dual role as a transcriptional activator of adipogenesis and an inhibitor of osteoblastogenesis, leading to a shift in the balance between fat formation and bone formation that favors adipogenesis over osteogenesis [4, 5, 6, 7, 8, 9]. While pioglitazone and rosiglitazone remain in clinical use under strict regulatory control, the bone loss associated with long‐term TZD use continues to be a major limitation of this drug class.

Our previous research identified a positive correlation between advancing age and the expression of zinc‐finger factor 521 (ZNF521) in human bone marrow mesenchymal stem cells (bmMSCs) [10]. ZNF521 was found to enhance adipogenic differentiation in mouse multipotent cells and aged human bmMSCs, primarily through upregulation of fatty acid synthase (FASN), a key enzyme in lipid biosynthesis [11, 12]. Knockdown of FASN effectively attenuated ZNF521's pro‐adipogenic effect, suggesting that targeting FASN activity could inhibit adipogenesis and potentially enhance osteoblastogenesis. Interestingly, TZDs such as troglitazone and netoglitazone (MCC‐555) were shown to inhibit ZNF521 expression and elicit a dual enhancing effect on both adipogenic and osteoblastic differentiation [13]. Structural studies revealed that the C3NS ring structure of MCC‐555 was critical for its dual effects, as derivatives lacking this feature failed to induce differentiation [13]. Furthermore, FASN knockdown selectively blocked the pro‐adipogenic effect of MCC‐555 while preserving pro‐osteoblastic activity [13]. These findings provide a foundation for exploring the combined use of TZDs and FASN inhibitors to optimize the osteogenic performance of aged bmMSCs.

(−)‐Epigallocatechin gallate (EGCG) is a major component of green tea. Recent findings suggest that EGCG can reduce oxidative stress and inflammatory cytokines in MSC cultures, supporting sustained osteogenesis in challenging environments [14]. EGCG is also a FASN inhibitor with reported anti‐obesity effects through inhibition of adipocyte differentiation [14, 15, 16, 17]. Mechanistically, EGCG inhibits FASN activity through a combination of reversible fast‐binding inhibition and irreversible slow‐binding inactivation [18]. EGCG has been integrated into natural and synthetic polymers, bioceramics, and hydrogels to promote osteogenesis and regulate macrophage polarization, which supports the potential of EGCG in bone regeneration applications [19]. Additionally, studies on pioglitazone‐loaded polylactic glycolic acid nanosphere composite scaffolds have demonstrated their efficacy in modulating the immune microenvironment to promote vascularization and bone regeneration [20, 21]. Building on these insights, our study examined the combined effects of pioglitazone and EGCG on adipogenic and osteoblastic differentiation in 2‐dimensional C3H10T1/2 cultures and the osteogenic performance of 3‐dimensional bmMSC cultures derived from an aged donor.

## Materials and methods

### Cell culture and maintenance

Mouse multipotent C3H10T1/2 clone 8 (RRID:CVCL_0190) was purchased from the American Type Cell Culture (ATCC, CCL‐226) in December 2022. Cells were cultured in low‐glucose Dulbecco's modified Eagle's medium (DMEM; Thermo Fisher Scientific, Waltham, MA, USA) supplemented with 10% fetal bovine serum (FBS; VWR International, Radnor, PA, USA), glutamine, penicillin, and streptomycin. Human bmMSCs, isolated from a 64‐year‐old male donor with informed consent (verbal), were approved for use by the Institutional Review Board of National Taiwan University Hospital, Hsin‐Chu Branch (IRB No. 103‐012‐F). The experimental protocols conformed to Declaration of Helsinki. These cells were maintained under similar conditions with 15% fetal bovine serum (Cytiva HyClone, Marlborough, MA, USA). Cultures were incubated at 37 °C in a humidified atmosphere with 5% CO_2_, and the medium was changed every 3–4 days. All experiments were performed with mycoplasma‐free cells. The reagents used included EGCG (Sigma‐Aldrich, Burlington, MA, USA), pioglitazone (Sigma‐Aldrich), oil red O (Sigma‐Aldrich), alizarin red S (Sigma‐Aldrich), cetylpyridinium chloride (CPC; Sigma‐Aldrich), KT5720 (Sigma‐Aldrich), and wortmannin (Sigma‐Aldrich). Antibodies targeting GSK3β, Ser9‐phosphorylated GSK3β, and β‐actin were obtained from Cell Signaling Technology (Danvers, MA, USA).

### Induction of adipogenic and osteoblastic differentiation

Adipogenic and osteogenic differentiation in C3H10T1/2 cells was induced as described [11]. Adipocyte differentiation was assessed by oil red O staining, followed by isopropanol destaining and absorbance measurement at 510 nm using a microplate reader (SpectraMax 190, Molecular Devices, San Jose, CA, USA). Osteogenic differentiation was evaluated by examining calcium precipitation, including alizarin red S staining, CPC destaining, and absorbance measurements at 595 nm using the same microplate reader, and by examining alkaline phosphatase expression using the staining kit (ab284936, Abcam, Cambridge, UK).

### Preparation of calcium‐alginate scaffolds and bioreactor system

Calcium‐alginate scaffolds and a continuous perfused bioreactor system were prepared as previously described [22, 23]. Human bmMSCs (5 × 10^6^ cells/10 scaffolds/treatment) were cultured for 1 day under static conditions before being transferred to the bioreactor for osteogenic induction at 37 °C and 5% CO_2_. The medium circulation was controlled by a peristaltic pump (G300‐2J, Longer, China) at 1 mL·min^−1^ for 7 days.

### Biomineralization and lipid accumulation analysis

Biomineralization and lipid accumulation in the cell clusters were analyzed using fluorescent staining. Mineralized areas were stained with xylenol orange (excitation/emission: 435/535 nm; Sigma‐Aldrich), whereas lipid droplets were stained with Nile red (excitation/emission: 488/575 nm; Sigma‐Aldrich). Hoechst 33342 (excitation/emission: 361/497 nm; Sigma‐Aldrich) was used for nuclear staining. High‐resolution images were acquired using a confocal microscope (LSM 900; Zeiss, Oberkochen, Baden‐Wurttemberg, Germany).

### Real‐time quantitative polymerase chain reaction

Total RNA from cells cultured in 2‐dimensional dishes was isolated using TRIzol reagent (Thermo Fisher Scientific). For RNA extraction from the bone‐like tissues (BLTs) embedded in calcium‐alginate scaffolds, the scaffolds were dissolved in 50 mm EDTA solution and BLTs were collected via brief centrifugation. Total RNA was purified using TRIzol reagent (Thermo Fisher Scientific). RT‐qPCR was performed using the KAPA SYBR® FAST One‐Step qRT‐PCR Master Mix kit (Sigma‐Aldrich) on a CFX 96 Real‐Time PCR System (Bio‐Rad, Hercules, CA, USA). The 5′ and 3′ primers used were as follows: mouse *Pparγ2*, TCGCTGATGCACTGCCTATG and GAGAGGTCCACAGAGCTGATT; and mouse *β‐Actin* (as a normalizer), CCCTGGCACCCAGCAC and GCCGATCCACACGGAGTAC. Human *RUNX2*, TTTGCACTGGGTCATGTGTT, and TGGCTGCATTGAAAAGACTG; human *alkaline phosphatase* (*ALP*), GACCCTTGACCCCCACAAT, and GCTCGTACTGCATGTCCCCT; human *BMP2*, TGTATCGCAGGCACTCAGGTCA, and CCACTCGTTTCTGGTAGTTCTTC; human *COL1A1*, GATTCCCTGGACCTAAAGGTGC, and AGCCTCTCCATCTTTGCCAGCA; and human *β‐ACTIN* (as a normalizer), AAGTCCCTTGCCATCCTAAAA, and ATGCTATCACCTCCCCTGTG. The relative mRNA expression levels were calculated using the $2^{-\Delta \Delta C_{T}}$ method.

### Analysis of mineral deposits by XRD and FT‐IR

The cell‐made biominerals were collected. To characterize the biomineral apatite, X‐ray diffraction (XRD; Miniflex II, Rigaku, Japan) was used to determine the crystalline structure, and Fourier transform infrared spectroscopy (FT‐IR; Spectrum 100, PerkinElmer, Shelton, CT, USA) was used to identify the functional groups.

### Statistical analysis of experimental data

All results were presented as the mean ± SD and were analyzed in triplicate. Statistical comparisons were performed using either the Student's *t*‐test, one‐way ANOVA, or two‐way ANOVA with Scheffe's *post hoc* test. Statistical significance was set at *P* < 0.05.

## Results and discussion

### Pioglitazone demonstrated dual adipogenic and osteogenic effects in 2‐dimensional cultures, highlighting its potential as an enhancer of osteogenesis

In bone marrow, bmMSC is the source of osteoprogenitor cells and osteoblasts. Any deficiency in the osteoblastogenic capacity of bmMSCs may lead to decline in bone formation activity in normal bone modeling and remodeling. Indeed, the deficiency of the osteoprogenitor cells to proliferate and differentiate into osteoblasts in aging and the decline in their capability to produce bone when implanted subcutaneously has been reported [24]. Deficiency in osteoprogenitor cells may also impact the repair of tissue damage and the recovery after surgical removal of diseased tissues. A satisfactory solution must be found to stimulate the impaired osteogenic lineage activity associated with aging. Our previous studies led us to examine the dual lineage effect of pioglitazone in multipotent cells to explore its pro‐osteogenic potential.

**Fig. 1:**
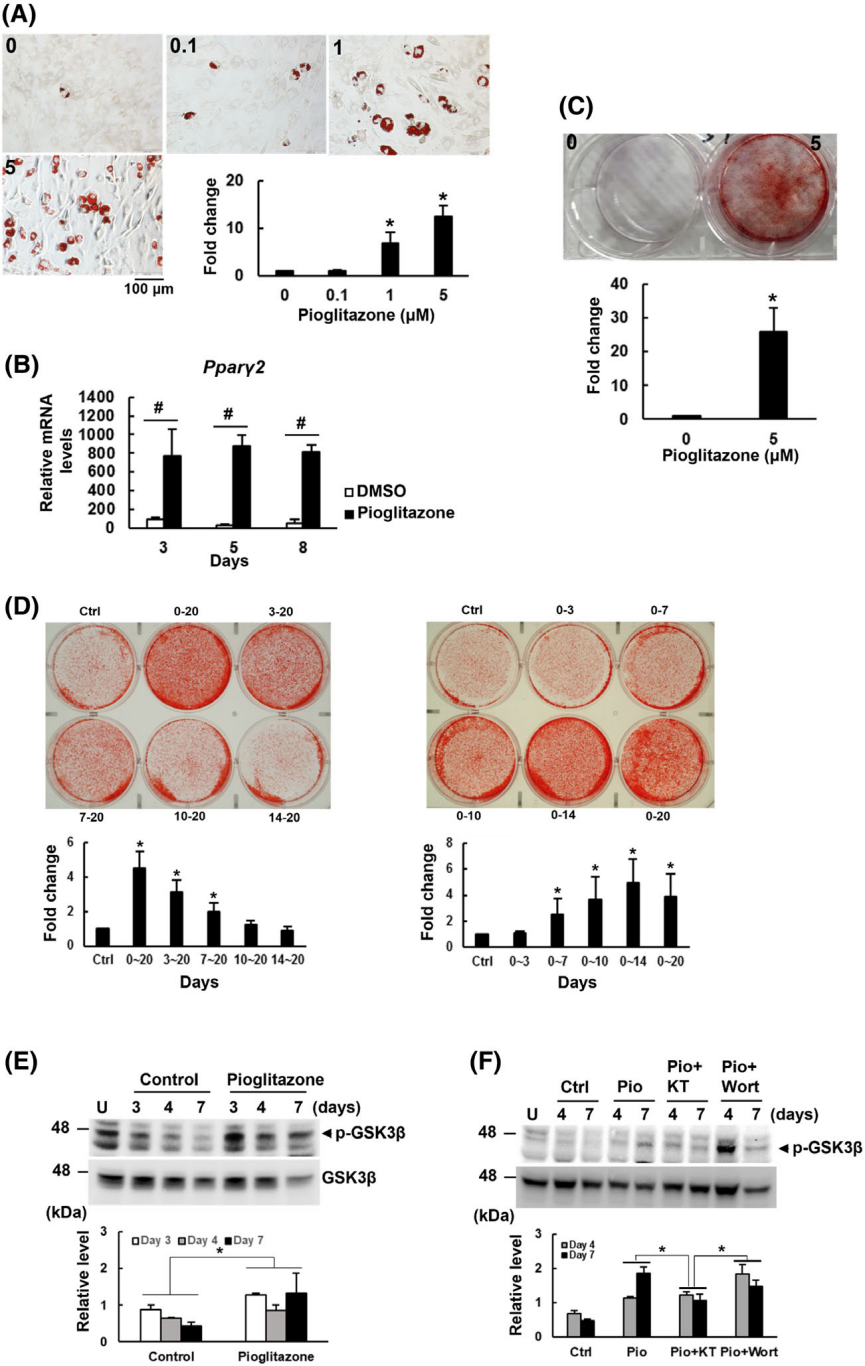
Effect of pioglitazone on the adipogenic and osteoblastic potentials of C3H10T1/2 cells. (A) Adipogenic induction. C3H10T1/2 cells were induced to the adipocytic lineage in the presence of 0, 0.1, 1, and 5 μm of pioglitazone. Cells were stained with oil red O at day 8. The stains were dissolved in propanol, quantitated, and compared with that of untreated control (to which a value of 1 was assigned). Comparison between the differentiation‐induced and uninduced (Day 0) was conducted by one‐way ANOVA with Scheffe's *post hoc* test. Data represent the means ± S.D. from three experiments (*n* = 3). *P < 0.05 versus untreated control. (B) RT‐qPCR analyses. C3H10T1/2 cells were induced to the adipocytic lineage for 0, 3, 5, and 8 days with concomitant treatment of the cells with either DMSO (as a control) or 5 μm of pioglitazone. The *Pparγ2* mRNA levels of cells harvested at day 3, day 5, and day 8 were normalized to that of day 0 (to which a value of 1 was assigned). Comparison between the differentiation‐induced and uninduced (Day 0) was conducted by one‐way ANOVA with Scheffe's *post hoc* test. Comparison between the control and pioglitazone‐treated at each time point was conducted by Student's *t*‐test. Data represent the means ± SD from three experiments. ^#^ *P* < 0.0001. (C) Osteoblastic induction. C3H10T1/2 cells were induced to the osteoblastic lineage for 14 days with or without co‐treatment of 5 μm of pioglitazone. Cells were stained with alizarin red S. The stains were quantitated, and the stain of pioglitazone‐treated cells was compared with that of the control cells (to which a value of 1 was assigned). Comparison between the pioglitazone‐treated and untreated was conducted by Student's *t*‐test. Data represent the means ± SD from three experiments. *P < 0.05. (D) Osteoblastic induction. Confluent C3H10T1/2 cells were induced for osteoblastic differentiation with 5 μm pioglitazone added at specific time points (days 0, 3, 7, 10, and 14; left) or at designated intervals (days 0–3, 0–7, 0–10, 0–14, and 0–20; right). On day 20, the cells were stained with alizarin red S to assess calcium deposition. Representative images are shown. Quantified stain intensities were normalized to that of the untreated control cells (Ctrl, assigned a value of 1). Data are presented as the mean ± SD of three experiments and were analyzed using one‐way ANOVA with Scheffe's *post hoc* test (*P < 0.05). (E) GSK3β phosphorylation (time course). Western blotting analysis of Ser9‐phosphorylated GSK3β was performed on cells induced for osteoblastic differentiation for 3, 4, and 7 days, with or without 5 μm pioglitazone. Phosphorylation signals were normalized to total GSK3β and compared with untreated control (U, assigned a value of 1). Data are shown as the mean ± SD from three experiments analyzed via two‐way ANOVA (*P < 0.005). (F) GSK3β phosphorylation (inhibitor studies). Western blotting analysis of Ser9‐phosphorylated GSK3β was conducted in cells induced for osteoblastic differentiation for 4 and 7 days in the presence of DMSO (Ctrl), 5 μm pioglitazone (Pio), pioglitazone plus 3 μm KT5720 (a PKA inhibitor; Pio + KT), or pioglitazone plus 3 μm wortmannin (a PI3K inhibitor; Pio + Wort). Phosphorylation signals were normalized to total GSK3β and compared with untreated control (U, assigned a value of 1). Data represent the mean ± SD from three experiments, analyzed using two‐way ANOVA with Scheffe's *post hoc* tests (*P < 0.05).

To evaluate the dual effects of pioglitazone, C3H10T1/2 cells were induced to undergo adipogenic differentiation, with or without concomitant treatment of the cells with varying concentrations of pioglitazone (0.1, 1, and 5 μm). Oil red O staining demonstrated a dose‐dependent increase in lipid droplet formation (*P* < 0.05, Fig. 1A). RT‐qPCR analysis further revealed significant upregulation of *Pparγ2* mRNA expression in the control (DMSO) and pioglitazone‐treated cells (*P* < 0.05), with pioglitazone eliciting stronger enhancing effect at all time points examined (Fig. 1B). These data demonstrate the strong adipogenic effect of pioglitazone. Based on these data, 5 μm of pioglitazone was chosen for subsequent osteoblastic differentiation experiment. The data demonstrate that pioglitazone significantly enhanced calcium deposition, as evidenced by alizarin red S staining (*P* < 0.05, Fig. 1C). These results indicate that pioglitazone promotes both adipogenesis and osteoblastogenesis, supporting its potential role in bone tissue engineering.

We investigated the timing of pioglitazone administration during osteoblastic differentiation to address its pro‐osteogenic mechanism. C3H10T1/2 cells co‐treated with 5 μm pioglitazone from day 0 to day 20 exhibited a 4.5‐fold (*P* < 0.05) increase in calcium deposition compared with untreated controls, whereas the calcium deposition was only 3.1 fold (*P* < 0.05) and 2 fold (*P* < 0.05) of the untreated control when pioglitazone was added at day 3 and day 7 post‐induction, respectively (Fig. 1D, left). In contrast, pioglitazone treatments initiated 10 days after osteoblastic induction failed to enhance calcium deposition, indicating a distinct timing dependency in pioglitazone's pro‐osteoblastic effect. Notably, restricting the pioglitazone co‐treatment to days 0–7, 0–10, 0–14, but not days 0–3, resulted in enhanced calcium deposition by 2.5 fold (*P* < 0.05), 3.7 fold (*P* < 0.05), and 4.9 fold (*P* < 0.05), respectively (Fig. 1D, right), emphasizing the critical role of early pioglitazone exposure (days 3–7) during the osteogenic commitment phase. In addition to calcium precipitation, we also examined the expression of alkaline phosphatase (ALP), whose expression is upregulated in the early phase of osteogenesis and is downregulated with the progression of mineralization. The results showed that short‐term pioglitazone co‐treatment, such as 0–3, 0–7, 7–17, 10–17 and 14–17 intervals, supported ALP expression, whereas longer pioglitazone co‐treatment downregulated ALP expression (Fig. S1). Taken together, these results indicate that pioglitazone can effectively prepare cells for osteoblastic differentiation during the initial induction phase, highlighting the importance of targeting this early window for mechanistic investigations.

The canonical Wnt/β‐catenin pathway plays a regulatory role in bone formation. Binding of Wnt ligand to its receptor results in the phosphorylation of glycogen synthase kinase 3β (GSK3β) at its serine 9 (Ser9) residue, which inactivates GSK3β and stabilizes β‐catenin. The cytosolic β‐catenin then translocates into nucleus to transactivate osteoblastogenic program [25]. Here, based on the timing dependency in pioglitazone's pro‐osteoblastic effect, C3H10T1/2 cells were induced to the osteoblastic lineage without or with pioglitazone co‐treatment for 3, 4, and 7 days for mechanistic studies. Western blotting analyses showed that the levels of Ser9‐phosphorylated GSK3β in the control cells decreased with treatment time, paralleling with their limited osteoblastic potential (Fig. 1E). The Ser9‐phosphorylated GSK3β signals in cells co‐treated with pioglitazone on days 3, 4, and 7 were 1.44, 1.36, and 3.01 fold, respectively, of those of the counterpart control cells. Statistical analyses indicated that pioglitazone significantly induced GSK3β phosphorylation (*P* < 0.005), where the role of treatment period was insignificant (*P* = 0.478). These data suggest that pioglitazone might promote osteoblastogenesis at least through the canonical Wnt/β‐catenin signaling pathway. Since protein kinase A (PKA) and phosphatidylinositol 3‐kinase (PI3K) phosphorylate GSK3β at Ser9, we examined if KT5720, a PKA inhibitor, and wortmannin, a PI3K inhibitor, counteract pioglitazone's effect on GSK3β phosphorylation. The results showed in general that pioglitazone increased GSK3β phosphorylation (*P* < 0.05), that KT5720 counteracted pioglitazone's activity (*P* < 0.05), whereas wortmannin failed to do so, and that there was a significant difference between the KT5720 and wortmannin co‐treatments (*P* < 0.05) (Fig. 1F). Thus, the observed co‐related timing dependency in calcium precipitation and the critical molecular processes involved in osteogenic commitment, particularly the inactivation of GSK3β activity by PKA, supports PKA as a major mediator of pioglitazone's pro‐osteoblastic effect. Since PKA activity is regulated by cyclic AMP (cAMP), which, in turn, is regulated by adenosine receptors, our findings imply that pioglitazone might interact with adenosine receptors to inactivate GSK3β activity via cAMP/PKA signaling. Further investigation on the interplay between pioglitazone and adenosine receptors might reveal more detailed mechanism behind pioglitazone's pro‐osteoblastic effect.

### EGCG selectively modulated pioglitazone‐induced differentiation by preserving osteogenesis and suppressing adipogenesis in 2‐dimensional C3H10T1/2 cultures

**Fig. 2:**
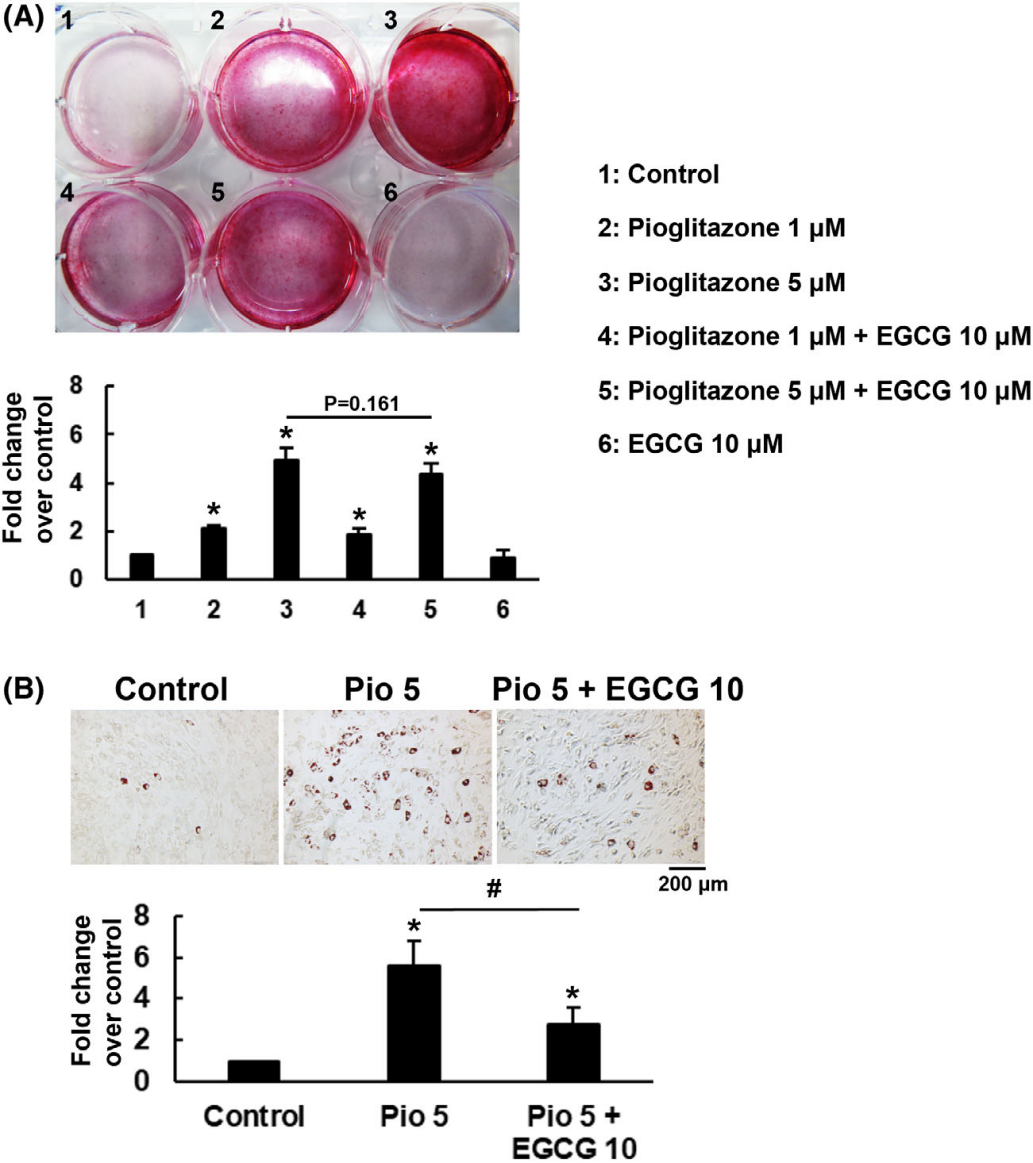
Effect of EGCG on the dual enhancing activity of pioglitazone in C3H10T1/2 cells. (A) Osteoblastic differentiation. C3H10T1/2 cells were induced into the osteoblastic lineage and treated concomitantly with pioglitazone (1 or 5 μm) and EGCG (10 μm), as indicated. On day 14, the cells were stained with alizarin red S to assess calcium deposition. The staining intensity was quantified and normalized to that of the untreated Ctrl cells (assigned a value of 1). Data (*n* = 3) were analyzed using one‐way ANOVA, and significant differences were noted (*P < 0.05). (B) Adipogenic differentiation. C3H10T1/2 cells were induced into the adipocytic lineage and treated concomitantly with 5 μm pioglitazone (Pio5) or pioglitazone in combination with 10 μm EGCG (Pio5 + EGCG10). On day 8, the cells were stained with oil red O to visualize lipid droplet formation. The staining intensity was quantified and normalized to that of untreated control cells (assigned a value of 1). Data were analyzed using one‐way ANOVA (*P < 0.05). Pairwise comparison between Pio5 and Pio5 + EGCG10 was made using Student's *t*‐test. Data represent the means ± SD from three experiments (*n* = 3).

Our findings emphasize the value of pairing pioglitazone with complementary agents to enhance its osteogenic efficacy while mitigating its pro‐adipogenic effect. Previous studies have shown that combining netoglitazone with FASN knockdown could selectively suppress adipogenesis without compromising osteogenic outcomes [13]; therefore, we explored the combining effect of pioglitazone and EGCG on the adipogenic and osteoblastic differentiation in C3H10T1/2 cells. We induced C3H10T1/2 cells to undergo osteoblastic differentiation with or without concomitant treatment of the cells with pioglitazone (1 and 5 μm), EGCG (10 μm), and pioglitazone plus EGCG for 14 days. Our data demonstrated that the calcium deposition activities of cells treated with 1 and 5 μm pioglitazone were approximately 2.1 (*P* < 0.05) and 4.9 (*P* < 0.05) fold of that of the control cells (Fig. 2A). EGCG co‐treatment maintained this osteogenic enhancement without antagonistic effects (*P* = 0.161). Conversely, while 5 μm pioglitazone markedly increased lipid droplet formation (5.6 fold, *P* < 0.05, Fig. 2B), EGCG significantly suppressed this pro‐adipogenic effect by reducing lipid accumulation (2.8 fold, *P* < 0.05). These findings establish EGCG as a selective modulator that mitigates pioglitazone‐induced adipogenesis while preserving osteogenic potential.

### Pioglitazone and EGCG cooperatively enhanced osteogenic differentiation and biomineralization in aged human bmMSCs

Although 2‐dimensional culture system provides foundational insights regarding the pro‐osteogenic effect of pioglitazone and EGCG dual‐treatment, extending these findings to 3‐dimensional culture systems is crucial for translating this approach into clinical practice. To explore the effects of pioglitazone and EGCG on osteogenesis, a 3‐dimensional cellular system was used. Human bmMSCs were cultured in calcium‐alginate scaffolds, which mimicked *in vivo* microenvironment, and maintained under osteoblastogenic induction in a constantly perfused bioreactor for 7 days (Fig. S2A). Scanning electron microscopy (SEM) revealed that in the EGCG‐treated (E) scaffolds, bmMSCs predominantly adhered to the scaffold surfaces, forming dispersed monolayer‐like structures without significant clustering, whereas in the pioglitazone‐treated (P) and PE dual‐treated scaffolds, bmMSCs formed compact, spherical aggregates (Fig. S2B).

**Fig. 3:**
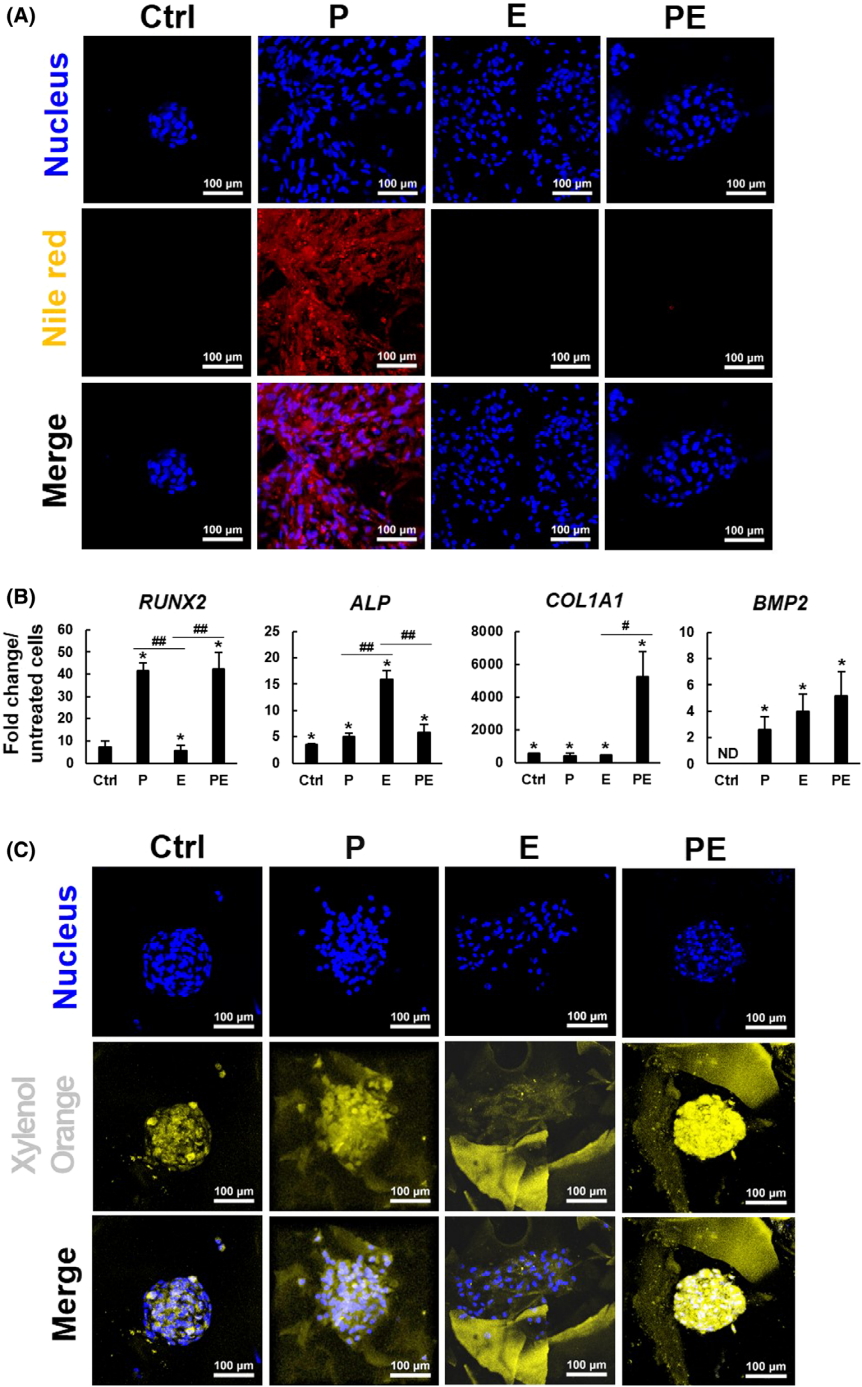
Biomineralization and lipid accumulation in cell clusters within scaffolds. (A) Lipid droplet formation. Ten scaffolds of the control, P‐, E‐, and PE‐treated groups (*n* = 10) were retrieved from the cell tanks 7 days after osteoblastic induction and were stained with Nile red (red) to detect lipid droplets. Representative images illustrate lipid droplet distribution and intensity. The size of the scale bar is 100 μm. (B) RT‐qPCR analysis. Total RNA was extracted from scaffolds 7 days after osteoblastic induction in the bioreactor. The expression levels of *RUNX2*, *ALP*, *COL1A1*, and *BMP2* were quantified and normalized to that of *β‐Actin* (as an internal control). Normalized mRNA levels were compared to those of 2‐dimensional bmMSC cultures before scaffold transfer (assigned a value of 1). Data represent mean ± SD from three experiments, analyzed using one‐way ANOVA with Scheffé's *post hoc* test (*P < 0.05). Pairwise comparisons were made using Student's *t*‐test (#*P* < 0.01; ##*P* < 0.005). (C) Biomineralization. Ten scaffolds of the control, P‐, E‐, and PE‐treated groups (*n* = 10) were stained with xylenol orange (yellow) to visualize biomineralized areas, and with Hoechst 33342 (blue) to localize the cell nuclei. Representative images show the distribution and intensity of biomineralization under different treatment conditions: Control (osteoblastic induction only, Ctrl), 5 μm pioglitazone (P), 10 μm EGCG (E), or their combination (PE). The size of the scale bar is 100 μm.

Nile red staining labeling the lipid droplets in the scaffolds showed that pioglitazone induced profound adipogenesis, whereas EGCG blocked adipogenesis (Fig. 3A), which is consistent with the data described in Fig. 2B. Then, we performed RT‐qPCR analyses to examine the expression of key osteogenic markers in the scaffolds. *RUNX2*, an early marker critical for initiating osteoblastic differentiation, was significantly upregulated in the P‐, E‐, and PE‐treated groups (*P* < 0.05), with P‐ and PE dual‐treatment showing the most pronounced expression (Fig. 3B). These data support the role of pioglitazone in promoting early lineage commitment. In contrary, *ALP*, a marker associated with extracellular matrix (ECM) maturation and mineralization, was significantly upregulated in the control, P‐, E‐, and PE‐treated groups (*P* < 0.05), and exhibited peak expression in the E‐treated group. These data support the notion that EGCG may participate in the mid‐stage of osteogenesis, while P‐ and PE dual‐treatment may support the coordinated progression of differentiation from early to mid‐stage. *COL1A1* is a mid‐to‐late marker of osteogenesis encoding type I collagen (COL1A1). In bones, COL1A1 serves as a crucial structural framework for cell adhesion, aggregation, and biological apatite deposition [26]. Our data showed that *COL1A1* was significantly upregulated in the control, P‐, E‐, and PE‐treated groups (*P* < 0.05), and PE dual‐treatment potently amplified this effect, suggesting that PE dual‐treatment might enhance the biological apatite‐binding capacity of the ECM and promote robust biomineralization [27]. Pioglitazone treatment did not enhance *COL1A1* expression as potently as PE treatment did, implying the requirement of complementary effects of EGCG and pioglitazone for maximum *COL1A1* induction. *BMP2*, a late‐stage marker critical for bone formation and tissue remodeling [28], was not detected in the control group, but was significantly induced by P‐, E‐, and PE treatments. *BMP2* showed the highest expression in the PE‐treated group, underscoring the ability of PE dual‐treatment to sustain differentiation into the final stage of osteogenesis. These findings support the notion that pioglitazone and EGCG may cooperatively influence the temporal cascade of osteogenic marker expression, driving bmMSCs through the early, mid, and late stages of osteoblastic differentiation.

Subsequently, xylenol orange staining labeling the calcified tissues in the scaffolds revealed that only sporadic biomineralization was observed in the cell clusters of the control scaffolds (Fig. 3C). In comparison, biomineralization was observed all over the cell aggregates in the P‐treated scaffolds, and the PE‐treated scaffolds exhibited more extensive biomineralization localized within cell aggregates. These data are consistent with the results of RT‐qPCR analyses. Notably, the surrounding scaffold surfaces of the E‐, P‐, and PE‐treated groups, but not the control group, also showed yellowish stains, suggesting that all the co‐treatments, including EGCG, enhanced the bmMSCs to produce and secrete biominerals. However, only sporadic biomineralization was observed in the cell clusters of the E‐treated scaffolds. Based on the observation that the E‐treated cells did not form aggregates (Fig. S1B), and that EGCG did not induce potent *COL1A1* expression, it is possible that the biomineral‐binding activity in the ECM of the E‐treated cells may be weaker than in the ECM of the P‐ and PE‐treated cells. The pronounced biomineralization in the PE‐treated group reflects a cooperative enhancement of osteoblastic differentiation and mineral deposition driven by the combined effects of pioglitazone and EGCG.

Our data that EGCG enhanced biomineral production in the 3‐dimensional human bmMSC cultures indicates EGCG as a pro‐osteoblastic agent. However, under our experimental design, EGCG alone did not significantly enhance calcium deposition within 14 days on the 2‐dimensional C3H10T1/2 cultures (Fig. 2A). It is imaginable that different types of cells may respond to a treatment with differential potency and timing. Given the reports that EGCG promoted osteoblastic differentiation in both human and mouse stem cells under prolonged exposure [29, 30, 31, 32], it is possible that further extension of the EGCG treatment time might be able to enhance mineralization in the 2‐dimensional C3H10T1/2 cultures. Notably, our data may present in advance that the 3‐dimensional culture system, which mimics *in vivo* microenvironment and allows aggregate formation, is superior to the 2‐dimensional culture system in deciphering the pro‐osteogenic effect of EGCG, pioglitazone, and their dual‐treatment.

**Fig. 4:**
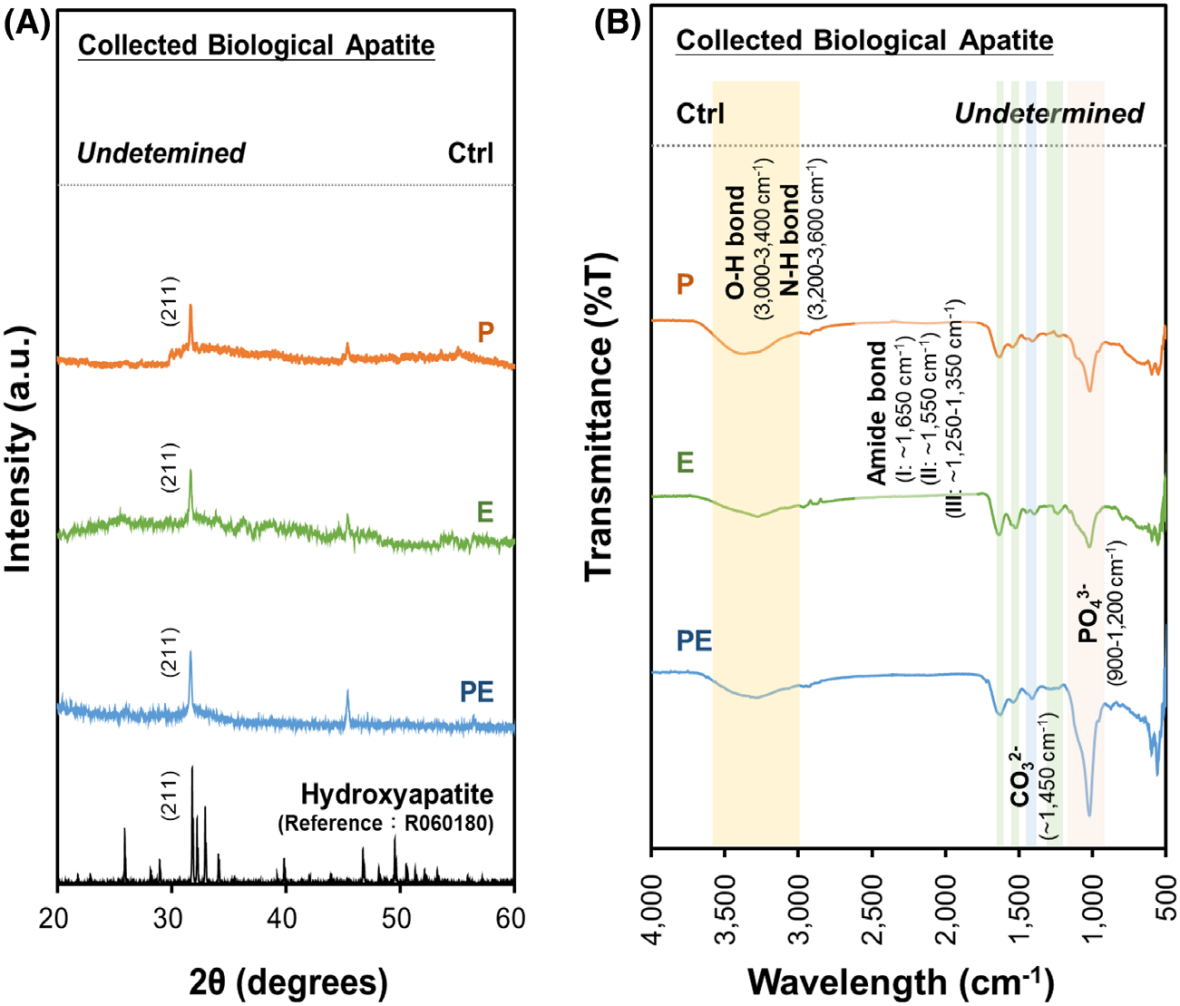
Characterization of the biological apatite secreted by 3‐dimensional bmMSC cultures in the bioreactor. (A) XRD analysis. After 7 days of incubation in the bioreactor, precipitates from the cell culture bottles were collected by centrifugation and air‐dried. The crystalline structure of the precipitates was analyzed using XRD with biological apatite as the standard reference. Representative diffraction patterns are shown. The characteristic (211) plane indicates the presence of biological apatite. (B) FT‐IR spectroscopy. The biological apatite precipitates were subjected to FT‐IR analysis to identify the key functional groups of biological apatite. Signals corresponding to phosphate (1200–900 cm^−1^), carbonate (~ 1450 cm^−1^), and hydroxyl (~ 3600–3000 cm^−1^) groups were detected, confirming the secreted biological apatite as bone‐like tissues.

Next, X‐ray diffraction (XRD) analyses on the cell‐made biominerals showed in the samples of the P‐, E‐, and PE‐treated groups a specific peak at (211) plane, a pattern matching to the diffraction pattern of hydroxyapatite (Fig. 4A). Parallel analyses using Fourier transform infrared (FT‐IR) spectroscopy revealed the existence of phosphate (around 1200–900 cm^−1^), carbonate (around 1450 cm^−1^), and hydroxyl (around 3600–3000 cm^−1^) signals only in the P‐, E‐, and PE‐treated groups, indicating that those calcified materials contained biological hydroxyapatite (Fig. 4B). Notably, the PE‐treated group exhibited the strongest biological apatite signals, characterized by the prominent phosphate and carbonate peaks. Thus, our data support the PE combination as a potent enhancer of the bone‐forming capability in aged bmMSCs.

Previously, using the 3D culture and bioreactor system, we have successfully enhanced the bone‐forming activity in the bmMSCs harvested from different aged donors for the preparation of bone‐like tissues [13, 23]. These results, together with current findings, highlight the consistency of this system in promoting the production of bone‐like tissues in the aged bmMSCs. Given the deleterious effect of age on the osteoblastic potential of bmMSCs, the quality and quantity of the bone‐like tissues produced by the bmMSCs may inversely proportional to the age of the donors from whom the bmMSCs are harvested. However, the use of potent osteogenic inducers might be able to minimize this age effect. In this study, we report that the PE dual treatment could be a potential candidate for enhancing the osteogenic capability in the aged bmMSCs for the preparation of bone‐like tissues.

Our data demonstrate the ability of PE dual treatment to activate the osteogenic capability in the aged bmMSCs *in vitro*, which might involve the complementary roles of pioglitazone and EGCG, including the inactivation of cellular GSK3β and stimulation of ECM production by pioglitazone, and selective suppression of adipogenesis by EGCG. It is worthy to note that in this study, only one dose of pioglitazone (5 μm) and EGCG (10 μm) was tested. To further elucidate how the PE treatment selectively promotes osteoblastogenesis, it would be necessary to adjust the doses of these drugs and examine if the doses and the ratio between these drugs affect the outcomes. Despite the *in vitro* findings, whether the PE dual treatment can modulate GSK3β signaling and enhance the osteogenic capability of bmMSCs *in vivo* is currently unknown. Several factors limit the validation of the osteogenic effect of this dual‐treatment *in vivo*. The doses and routes of drug administration and the rates of drug absorption and elimination may affect the blood concentration of pioglitazone and EGCG, disrupting their cooperative activity. BmMSCs is a small population of cells in the bone marrow, it is difficult to locate them and to evaluate the change in their osteogenic capability *in vivo*. Besides, unlike the *in vitro* experiments where only bmMSCs are studied, the systemic effect of the PE treatment might elicit off‐target effects.

The bioreactor system opens a window for exploiting the clinical potential of our PE dual‐treatment strategy. The bioreactor system provides continuous medium perfusion to ensure consistent nutrient delivery and waste removal, preventing hypoxic conditions that can cause cell death and impede differentiation. Additionally, it has been shown that the medium flow in the bioreactor system may activate mechanotransduction pathways that further enhance osteogenic differentiation and matrix mineralization [33]. The bioreactor system enables us to use the PE‐treated aged bmMSCs to produce large scale of bone‐like tissues. Thus far, orthopedic surgery is a conventional way to treat osteoporotic fractures. Elderly patients often experience delayed healing due to reduced bone regeneration capacity. Bone grafts, including autografts, allografts, alloplastic grafts, and xenografts, are commonly used to facilitate recovery, especially for treating severe large‐scale injuries. However, the use of these grafts is confronted by donor site complications, infections, limited tissue availability, immune rejection, and low bioavailability. While the PE dual treatment and the bioreactor enable large‐scale production of autologous bone‐like tissues from the 3‐dimensional bmMSC cultures, future studies using bone injury animal models are needed to examine if the cell‐made bone‐like tissues can serve as bone grafts to facilitate the healing process. A comparative study using the bmMSCs harvested from a group of aged donors is also required to evaluate the potency of the PE dual‐treatment in enhancing their bone‐forming capability. These data may pave the way for future evaluating the application of our PE dual‐treatment strategy in bone tissue engineering in clinical trials.

## Conflict of interest

The authors declare that no competing interest exists.

## Author contributions

C‐YC: methodology design, supervision, and validation of the experiments; J‐CJ, C‐YP, and QCN: investigations; F‐HL and H‐MW: conceptualization of the study; SL: conceptualization, supervision, methodology design, and manuscript preparation. All authors read and approved the final manuscript version.

## Supporting information


**Fig. S1.** Osteoblastic induction.
**Fig. S2**. Osteoblastic induction in the 3‐dimensional bmMSC cultures.

## Data Availability

The data that support the findings of this study are available from the corresponding author [cshe1534@csh.org.tw] upon reasonable request.
