# Supplementary material for: Pioglitazone plus (−)‐epigallocatechin gallate: a novel approach to enhance osteogenic performance in aged bone marrow mesenchymal stem cells
Source: FEBS Open Bio. 2025 Dec 5;16(5):932–43. doi: 10.1002/2211-5463.70175 (PMC13145338; doi:10.1002/2211-5463.70175)
Supplement: Supplementary file 1 — Fig. S1. Osteoblastic induction. Fig. S2. Osteoblastic induction in the 3‐dimensional bmMSC cultures. [file FEB4-16-932-s001.zip › Figures_legends.docx]

**Supporting Information**

**Supplementary Fig. S1.** Osteoblastic induction.

**Supplementary Fig. S2.** Osteoblastic induction in the 3-dimentional bmMSC cultures.
