# Supplementary figures and images for: Pioglitazone plus (−)‐epigallocatechin gallate: a novel approach to enhance osteogenic performance in aged bone marrow mesenchymal stem cells
Source: FEBS Open Bio. 2025 Dec 5;16(5):932–43. doi: 10.1002/2211-5463.70175 (PMC13145338; doi:10.1002/2211-5463.70175)

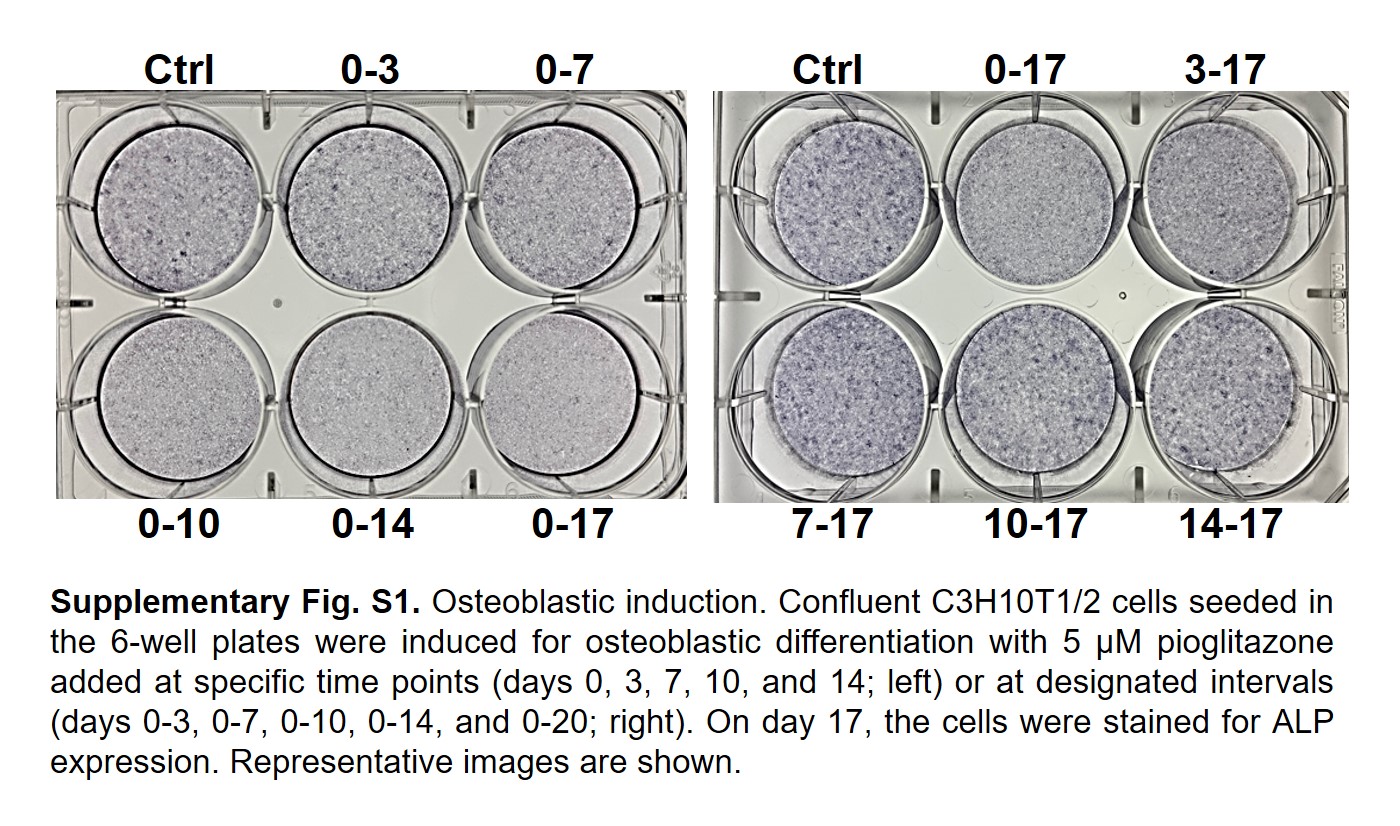

Supplement: Supplementary file 1 — Fig. S1. Osteoblastic induction. Fig. S2. Osteoblastic induction in the 3‐dimensional bmMSC cultures. [file FEB4-16-932-s001.zip › feb470175-sup-0001-FigureS1.jpg]

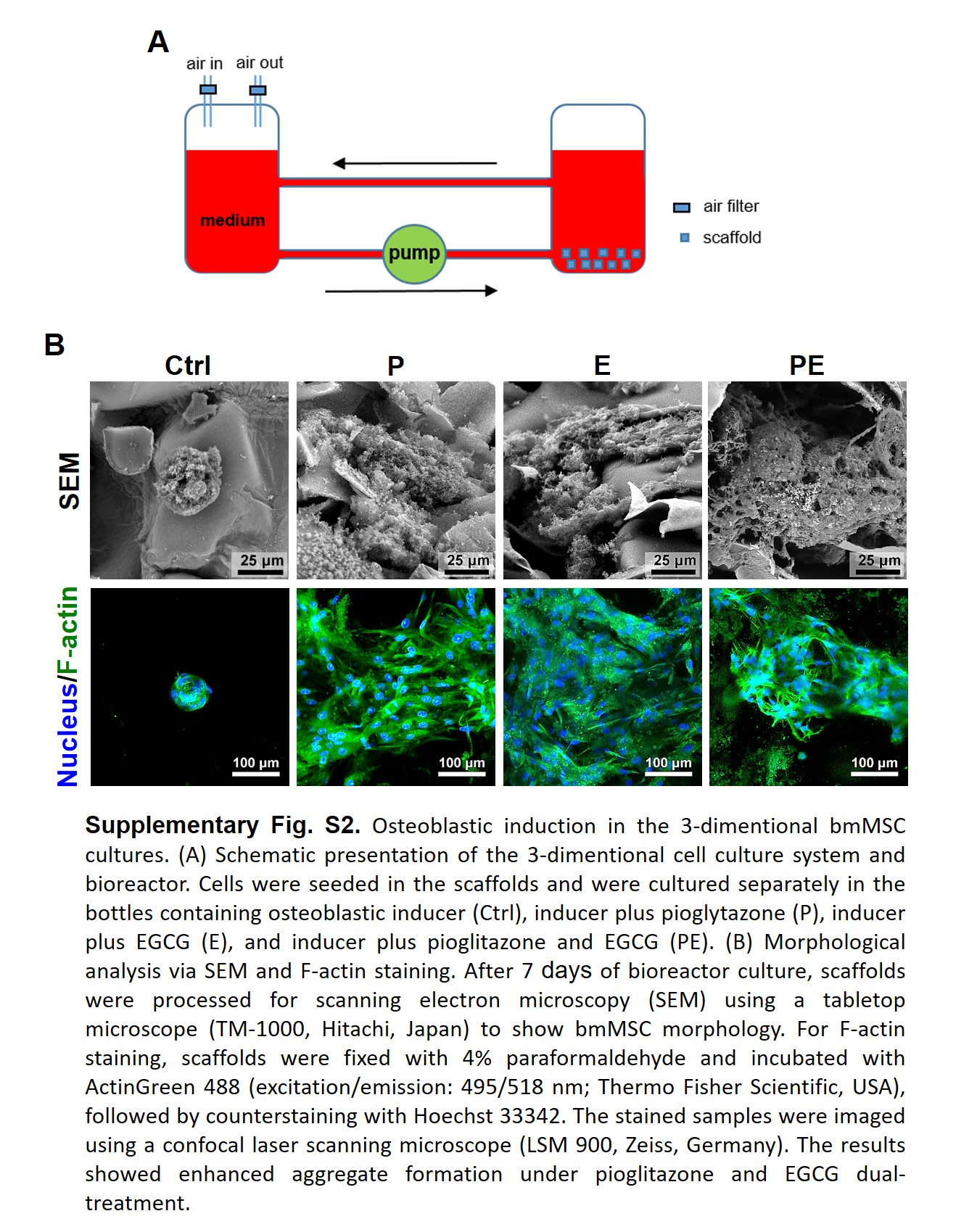

Supplement: Supplementary file 1 — Fig. S1. Osteoblastic induction. Fig. S2. Osteoblastic induction in the 3‐dimensional bmMSC cultures. [file FEB4-16-932-s001.zip › feb470175-sup-0002-FigureS2.jpg]
